# Cancer History and Systemic Anti-Cancer Therapy Independently Predict COVID-19 Mortality: A UK Tertiary Hospital Experience

**DOI:** 10.3389/fonc.2020.595804

**Published:** 2020-11-20

**Authors:** Christopher C. T. Sng, Yien Ning Sophia Wong, Anjui Wu, Diego Ottaviani, Neha Chopra, Myria Galazi, Sarah Benafif, Gehan Soosaipillai, Rebecca Roylance, Alvin J. X. Lee, Heather Shaw

**Affiliations:** ^1^ Cancer Division, University College London Hospitals NHS Foundation Trust, London, United Kingdom; ^2^ UCL Cancer Institute, University College London, London, United Kingdom; ^3^ NIHR University College London Hospitals Biomedical Research Centre, London, United Kingdom

**Keywords:** COVID-19, SARS-CoV-2 infection, solid cancers, risk factors, systemic anti-cancer therapy, co-morbidity

## Abstract

**Background:**

The COVID-19 pandemic remains a pressing concern to patients with cancer as countries enter the second peak of the pandemic and beyond. It remains unclear whether cancer and its treatment contribute an independent risk for mortality in COVID-19.

**Methods:**

We included patients at a London tertiary hospital with laboratory confirmed SARS-CoV-2 infection. All patients with a history of solid cancer were included. Age- and sex-matched patients without cancer were randomly selected. Patients with hematological malignancies were excluded.

**Results:**

We identified 94 patients with cancer, matched to 226 patients without cancer. After adjusting for age, ethnicity, and co-morbidities, patients with cancer had increased mortality following COVID-19 (HR 1.57, 95% CI:1.04–2.4, *p* = 0.03). Increasing age (HR 1.49 every 10 years, 95% CI:1.25–1.8, *p* < 0.001), South Asian ethnicity (HR 2.92, 95% CI:1.73–4.9, *p* < 0.001), and cerebrovascular disease (HR 1.93, 95% CI:1.18–3.2, *p* = 0.008) also predicted mortality. Within the cancer cohort, systemic anti-cancer therapy (SACT) within 60 days of COVID-19 diagnosis was an independent risk factor for mortality (HR 2.30, 95% CI: 1.16–4.6, *p* = 0.02).

**Conclusions:**

Along with known risk factors, cancer and SACT confer an independent risk for mortality following COVID-19. Further studies are needed to understand the socio-economic influences and pathophysiology of these associations.

## Introduction

The Coronavirus disease 2019 (COVID-19) global pandemic caused by the SARS-CoV-2 virus necessitated urgent and pragmatic measures to protect the most vulnerable people in society, including those with cancer. The early narratives that patients with cancer may have poorer outcomes in COVID-19 were shaped predominantly by a small number of North American and Chinese studies comparing patients with cancer to those without cancer. These studies reported a high total case fatality rate for patients with cancer hospitalized with COVID-19, ranging from 11 to 28% (1–3). Established risk factors for severe COVID-19, such as increasing age and co-morbidity are common in these patients. However, it remains unclear whether cancer contributes an independent risk to mortality in COVID-19. In addition to patient factors, geographical variation in patient demographics and oncological treatment may contribute to significant heterogeneity of outcomes in patients with cancer who develop COVID-19 (4).

Concerns have also been raised that systemic anti-cancer therapy (SACT) may worsen outcomes in COVID-19. Moreover, timely management of cancer means exposure to potential drug side effects, including immunosuppression, and exposure to healthcare environments. This has led governing bodies to advise self-isolation or ‘shielding’ for vulnerable patients with cancer and prioritization of anti-cancer treatment (5, 6). However, accurate risk stratification remains hampered by a lack of detailed understanding of the pre-morbid and treatment-related risk factors. Multicenter projects such as the UK Coronavirus Cancer Monitoring Project (UKCCMP) (7) and the COVID-19 & Cancer Consortium which includes patients from the USA, Canada, and Spain (CCC19) (4) seek to provide insights into these concerns but do not make comparison with patients without cancer.

Here we explore the outcomes of patients with cancer compared to patients without cancer who were diagnosed with SARS-CoV-2 infection at a tertiary hospital in London, UK. The large cancer practice and acute medical care reflect the local demographic composition. We included patients from the early phase of SARS-CoV-2 spread in London, before the widespread availability of surveillance testing. Information was collected on cancer subtypes, cancer staging, and anti-cancer treatment to better understand their relative contributions to the risk of mortality from COVID-19. Additionally, with a dedicated COVID-19 follow-up service and ongoing regular outpatient oncology follow-up, we were able to capture longitudinal data for patients following discharge from hospital. This study adds valuable insights to aid policy-makers and healthcare professionals in minimizing risks to patients with cancer and providing the safe delivery of anti-cancer treatments.

## Materials and Methods

### Ethics

In view of the retrospective nature of this audit, approval from a Research Ethics Committee (REC) within the UK Health Departments Research Ethics Service and Health Research Authority (HRA) was not necessary.

### Data Collection

Anonymized retrospective data was collected from electronic health records of the University College London Hospitals NHS Foundation Trust. Patients aged 18 years or over with a laboratory confirmed SARS-CoV-2 on RT-PCR from a throat/nose swab between 1 March 2020 and 31 May 2020 were included in this study. Patients with a history of current or previously confirmed solid cancer were identified. Age- and sex-matched patients with no previous or current history of cancer were selected randomly for comparison. Patients with hematological malignancies were excluded from both groups. The primary outcome was all-cause mortality. Additional relevant data was collected for the cancer cohort. This included blood results at time of diagnosis of SARS-CoV-2 infection, COVID-19 symptoms, admission length, and cancer-specific data. Follow-up data was collected from 1 March 2020 until 12 June 2020.

### Definitions

Active cancer was defined as a cancer diagnosis or anti-cancer treatment within the last 12 months or radiological or biochemical evidence of active or recurrent cancer. Active anti-cancer therapy was defined as any anti-cancer therapy within 60 days of the COVID-19 diagnosis, including SACT, radiotherapy, and surgery. SACT included all systemic anti-cancer treatment modalities such as cytotoxic chemotherapy, endocrine therapy, immunotherapy, and targeted anti-cancer therapy.

### Statistical Analysis

Descriptive statistics was used to compare baseline demographic data between patient cohorts. Continuous data is presented as median and interquartile range (IQR). Categorical data is shown as frequency and percentage. To test the statistical significance, we performed Mann–Whitney U tests for continuous data and Fisher’s exact test or Chi-Square test for categorical variables.

Cox proportional-hazards regression model was used to study survival from the date of COVID-19 diagnosis. Univariate analysis was used to obtain crude hazard ratios for mortality following COVID-19 for each pre-morbid risk factor. Risk factors which were statistically significant at univariate analysis (age, South Asian ethnicity, cardiovascular disease, chronic kidney disease, hypertension, and cerebrovascular disease) were adjusted for in a multivariate model when assessing the independent impact of cancer history. In order to study recent anti-cancer treatment, subgroup analysis of the cancer cohort was performed. Significant variables at univariate analysis of the cancer cohort (age, hypertension, and cerebrovascular disease) were adjusted for in a multivariate model to elucidate the effect of anti-cancer treatments on overall survival within the cancer cohort. All survival analyses were conducted using Lifelines 0.24.2 in Python 3.6.4 and survival package on R 3.5.2. For blood investigations, box and violin plots were created and Mann-Whitney U tests were performed using GraphPad Prism version 8.3 for Mac (GraphPad Software, San Diego, California USA).

## Results

### Demographics of Patients With Cancer and Patients Without Cancer

626 patients with laboratory confirmed SARS-CoV-2 infection between 1 March 2020 and 31 May 2020 were identified. 94 (15.0%) patients had a history of cancer. 226 age- and sex-matched patients without cancer were selected randomly for comparison with the cancer cohort.

The combined median follow-up time from date of COVID-19 diagnosis (time to event or last clinical contact) for both cohorts was 18 days (IQR 7.8–44), during which 118 (36.9%) patients died. **Table 1** summarizes the demographic features of the cancer and non-cancer cohorts. The median time from COVID-19 diagnosis to death in the cancer cohort was similar to the non-cancer cohort (8 days (IQR 4–13) *vs* 7 days (IQR 4–15), *p* = 0.64).

**Table 1:** Demographic data of patients with SARS-CoV-2 infection. Data is shown as n (%) or median (IQR).

	Non-cancer	Cancer	*p*-value
Total	226	94	
Male	152 (67%)	62 (66%)	0.82
Female	74 (33%)	32 (34%)	–
Median age (years)	70.5 (60–80)	71 (62–80)	0.42
BMI (kg/m^2^)	26.6 (23.5–30.5)	25.1 (21.7–30.5)	<0.001
**Ethnicity**			
South Asian	28 (12%)	8 (9%)	0.25
Black	37 (16%)	6 (6%)	0.01
Other	19 (8%)	9 (10%)	0.83
White	115 (51%)	64 (68%)	0.01
Unknown	27 (12%)	7 (7%)	–
**Smoking status**			0.04
Lifetime non-smoker	110 (49%)	40 (43%)	–
Ex-smoker	58 (26%)	42 (45%)	–
Active smoker	13 (6%)	7 (7%)	–
Unknown	17 (8%)	4 (4%)	–
**Co-morbidities**			
Cardiovascular disease	59 (26%)	18 (19%)	0.18
Dementia	35 (15%)	7 (7%)	0.05
Diabetes	73 (32%)	24 (26%)	0.23
Congestive cardiac failure	16 (7%)	9 (10%)	0.45
Liver disease	4 (2%)	3 (3%)	0.43
Hypertension	123 (54%)	37 (39%)	0.01
Peripheral vascular disease	13 (6%)	2 (2%)	0.16
Cerebrovascular disease	37 (16%)	12 (13%)	0.41
Chronic lung disease	47 (21%)	14 (15%)	0.22
Chronic kidney disease	26 (12%)	12 (13%)	0.75
Ongoing corticosteroid therapy	13 (6%)	4 (4%)	0.59

BMI, body mass index.

There were differences between the cancer and non-cancer cohorts: the cancer cohort was comprised of more White patients (68 *vs* 51%, *p* = 0.01) and fewer Black patients (6 *vs* 16%, *p* = 0.01). The median BMI was lower in those with cancer (25.1 *vs* 26.6 kg/m^2^, *p* < 0.001). A greater proportion of patients with cancer were ex- or active smokers (52 *vs* 31%, *p* = 0.04). There was also a lower incidence of hypertension in the cancer cohort (39 *vs* 54%, *p* = 0.01).

### Clinical Features of SARS-CoV-2 Infection in Patients With Cancer

**Figure 1:**
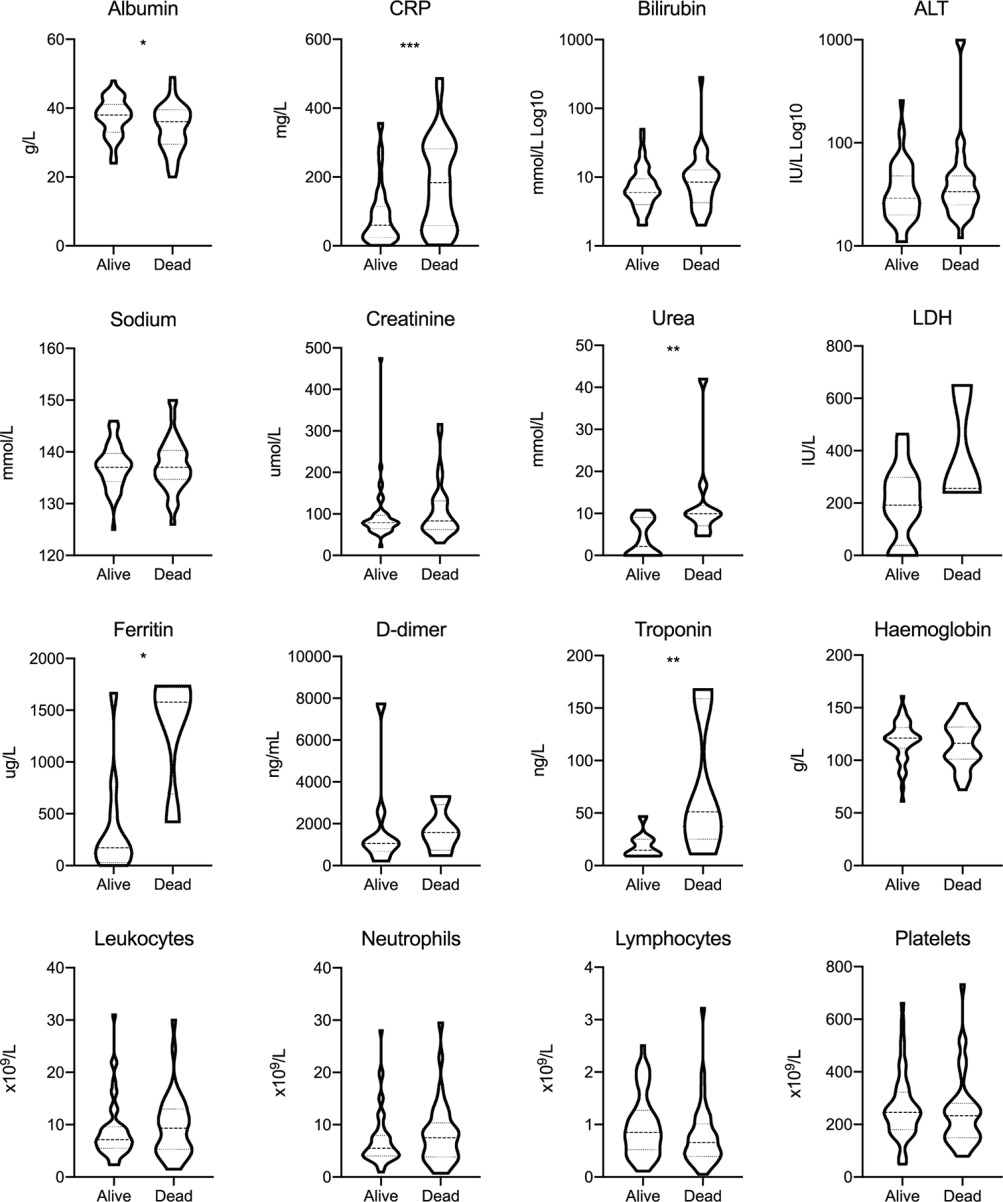
Blood investigations for patients with cancer at presentation with SARS-CoV-2 infection. Dashed lines represent the median and dotted lines represent the IQR. *p < 0.05; **p < 0.01; ***p < 0.001; CRP, C-reactive protein; ALT, alanine transaminase; LDH, lactate dehydrogenase.

The clinical features of COVID-19 in patients with cancer were consistent with existing reports (8). The most common symptoms at presentation for patients with cancer were cough (60.6%), fever (53.2%), and dyspnoea (41.5%). 33 (35.1%) patients had mild (not requiring oxygen) or asymptomatic illness. 22 (23.4%) of the patients with cancer were admitted to an intensive therapy unit (ITU) or high dependency unit (HDU), of which five (5.3%) were intubated and 13 (13.8%) received non-invasive ventilation including Continuous Positive Airway Pressure (CPAP). The ITU/HDU admission rate was similar for those with active cancer (22.4%). However, patients with metastatic disease had a significantly lower rate of ITU/HDU admission (5.3%, OR 0.14, 95% CI: 0.01–0.82, *p* = 0.03) compared to those with less advanced cancer. The incidence of suspected nosocomial SARS-CoV-2 infection was 37.2% in our cancer cohort, while the majority acquired SARS-CoV-2 infection in the community (61.7%; one missing data). Elevated C-reactive protein (CRP) (*p <*0.001), urea (*p* = 0.002), ferritin (*p* = 0.01), troponin (*p* = 0.009), and low albumin (*p* = 0.02) at presentation were predictors of mortality in the cancer cohort (**Figure 1**).

### General Risk Factors for COVID-19 Mortality

**Figure 2:**
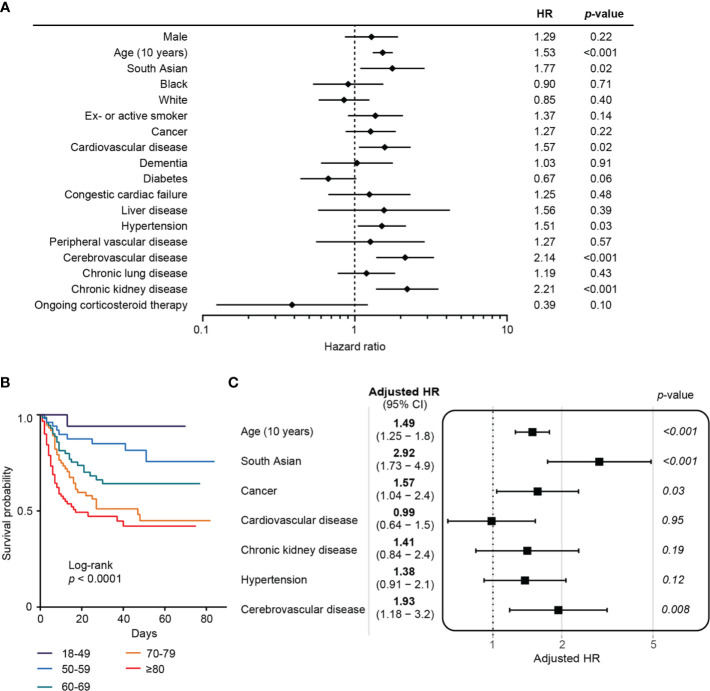
Risk factors for COVID-19 mortality in combined cancer and non-cancer cohorts. **(A)** Forest plot showing the hazard ratios from univariate analysis of risk factors associated with mortality in COVID-19. Horizontal bars indicate 95% CI. HR, hazard ratio. **(B)** Kaplan–Meier plot of survival analysis by age. **(C)** Multivariate analysis of risk factors associated with mortality following COVID-19.

Univariate analysis of the combined cancer and non-cancer cohorts demonstrated that increasing age, South Asian ethnicity, chronic kidney disease, cerebrovascular disease, cardiovascular disease, and hypertension were associated with increased mortality (**Figures 2A, B**).

At the time of analysis, a greater proportion of patients with cancer had died compared to those in the non-cancer cohort (43.6 *vs* 34.1%). This difference in mortality between cancer and non-cancer cohorts was statistically significant among those aged 70 years and above (OR 2.28, 1.14–4.50, *p* = 0.02). This trend was not seen when comparing those with and without cancer under 70 years old (OR 0.87, 0.36–2.10, *p* = 0.83). A multivariate survival analysis was performed to evaluate the independent contribution of cancer to mortality risk following SARS-CoV-2 infection, adjusting for age, ethnicity, and co-morbidities which were significant in the univariate analysis. A history of cancer was an independent risk factor for mortality (HR 1.57, 95% CI: 1.04–2.4, *p* = 0.03) (**Figure 2C**). Active cancer was associated with a similar adjusted risk for mortality in this model (HR 1.64, 95% CI: 1.03–2.6, *p* = 0.04). Other variables that remained significant in the multivariate survival analysis were increasing age (HR 1.49 for every 10 years, 95% CI: 1.25–1.8, *p* < 0.001), South Asian ethnicity (HR 2.92, 95% CI: 1.73–4.9, *p* < 0.001), and cerebrovascular disease (HR 1.93, 95% CI: 1.18–3.2, *p* = 0.008) (**Figure 2C**).

### Pre-Morbid Characteristics and Risk Factors for COVID-19 Mortality in Patients With Cancer

The most common cancer type in this cohort was genitourinary (n = 24), followed by gastrointestinal (n = 23), thoracic (n = 15), and gynecological cancers (n = 9). The most common histological type was adenocarcinoma (43.6%), followed by squamous cell carcinoma (17%). 58 (61.7%) patients from the cancer cohort had active cancer. There were no significant differences in mortality between cancer types or histological subtypes (**Supplementary Table 2**). 19 (20.2%) patients had metastatic disease at time of COVID-19 diagnosis. The sites of metastases, of which some patients had multiple, included soft tissue (n = 13), bone (n = 8), brain (n = 5), and extra-regional lymph nodes (n = 4). Overall, patients with metastatic disease were not found to have a higher mortality following COVID-19 (HR 1.13, 95% CI: 0.54–2.37, *p* = 0.75) (**Supplementary Table 2**).

### Systemic Anti-Cancer Therapy and Mortality in COVID-19

We next explored whether patients receiving SACT within 60 days of their COVID-19 diagnosis, were at greater risk of mortality from COVID-19. 25 (26.6%) patients were receiving SACT, including chemotherapy (n = 15), endocrine therapy (n = 8), immunotherapy (n = 4), and targeted anti-cancer therapy (n = 2). Four were receiving a combination of two types of SACT. 15 of these patients were being treated with palliative intent. One patient received only radiotherapy, and one patient received only surgery. Patients receiving SACT had greater incidence of metastatic disease (48.0 *vs* 10.6%, *p* < 0.001) and were younger (median age 62.5 *vs* 73.0 years, *p* = 0.01) compared to those not on therapy. They were also more likely to have anaemia (*p* = 0.04) and lymphopenia (*p* = 0.01).

**Figure 3:**
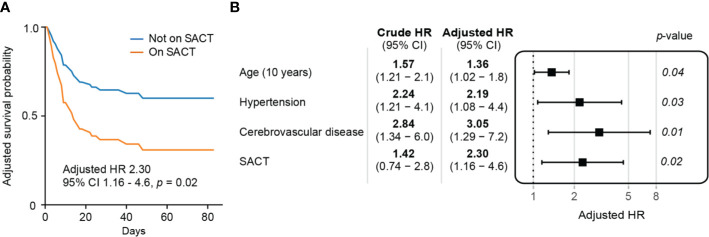
Assessing recent systemic anti-cancer therapy (SACT) as a predictor of mortality following COVID-19 **(A)** Adjusted survival curves of patients on systemic anti-cancer therapy with COVID-19. **(B)** Multivariate survival analysis of pre-morbid risk factors in patients with cancer and COVID-19.

In order to assess whether SACT was an independent risk factor for mortality following COVID-19, we performed multivariate survival analysis, adjusting for risk factors which were significant at univariate analysis in the cancer cohort (age, hypertension, and cerebrovascular disease) (**Supplementary Table 1**). Receipt of SACT was an independent risk factor for COVID-19 mortality in the cancer cohort (HR 2.30, 95% CI: 1.16–4.6, *p* = 0.02) (**Figures 3A, B**). Multivariate survival analysis of the individual treatment modalities was limited by small numbers but did not identify significant differences in COVID-19 mortality (**Table 2**).

**Table 2:** Multivariate survival analysis comparing recent types of anti-cancer treatment in patients with cancer and COVID-19, adjusted for age and co-morbidities (hypertension and cerebrovascular disease).

	HR (95% CI)	*p*-value
All systemic anti-cancer therapy	2.30 (1.16–4.6)	0.02
All chemotherapy	2.04 (0.84–4.9)	0.11
Palliative chemotherapy	2.29 (0.87–6.0)	0.09
Neoadjuvant/adjuvant chemotherapy	1.32 (0.30–5.9)	0.71
Endocrine therapy	1.94 (0.74–5.1)	0.18
Targeted anti-cancer therapy	3.44 (0.44–27.2)	0.24
Immunotherapy	1.70 (0.39–7.3)	0.48
Radiotherapy	1.94 (0.45–8.4)	0.38
Surgery	1.40 (0.18–10.8)	0.75

Increasing age (HR 1.36 for every 10 years, 95% CI: 1.02–1.8, *p* = 0.04), hypertension (HR 2.19, 95% CI: 1.08–4.4, *p* = 0.03), and cerebrovascular disease (HR 3.05, 95% CI: 1.29–7.2, *p* = 0.01) remained significant risk factors for mortality in this multivariate analysis (**Figure 3B**). A full breakdown of the cancer cohort’s demographics, co-morbidities, and univariate analysis of cancer-specific data are described in the appendices (**Supplementary Tables 1** and **2**).

## Discussion

This is one of the first UK/European retrospective cohort study of SARS-CoV-2 infection in patients with cancer and matched patients without cancer. 94 patients with cancer were compared to 226 matched patients without cancer at a London tertiary hospital. Risk factors for mortality were found to be consistent with current literature in that increasing age and a range of vascular co-morbidities (chronic kidney disease, cerebrovascular disease, cardiovascular disease, and hypertension) predicted mortality in COVID-19 (8, 9).

South Asian ethnicity was an independent risk factor for mortality in our cohort. Preliminary UK-wide data showed that hospitalized South Asians, but not Black patients, have a higher adjusted mortality in COVID-19 (10). Meanwhile, the primarily North American CCC19 did not find an association between ethnicity and COVID-19 mortality among patients with cancer (4), nor did a study of patients with cancer and COVID-19 in New York (2). This may be explained by different demographics and ethnicity classifications in North America. Both studies did not include a South Asian ethnicity category.

When accounting for age, ethnicity, and co-morbidities, cancer was an independent risk factor for mortality following COVID-19 in this study (HR 1.57, 95% CI: 1.04–2.4, *p* = 0.03). The reasons for this effect are likely multi-faceted, including cachexia, anemia, and abnormal immunity. Our findings show that hypo-albuminemia, which is linked to both poor nutrition and a pro-inflammatory state, is associated with mortality in the cancer cohort. Elevated levels of pro-inflammatory cytokines, including interleukin-6 (IL-6) have also been noted in patients with cancer and COVID-19 (3) and is linked to severe lung pathology and mortality in COVID-19 (11). IL-6 production within the tumor microenvironment could directly contribute to poorer outcomes in patients with cancer (12). Therapeutic modulation of IL-6 may therefore be particularly relevant to patients with cancer and COVID-19.

The association between cancer and COVID-19 mortality has been shown in previous cohort studies from China. A study of 105 patients with cancer from 14 hospitals in Hubei province, China, reported an odds ratio for mortality in COVID-19 of 2.34 (*p* = 0.03) (1). Another in Wuhan, China, found similar odds of death in 232 patients with cancer (OR 2.0, *p* = 0.001) (3). Two studies in New York had differing findings: A study of the Mount Sinai Health System (334 patients with cancer, 5,354 patients without cancer) showed that patients with cancer had a higher likelihood of intubation but no statistically significant difference in overall unadjusted mortality (RR 1.15, 95% CI: 0.84–1.57) (13). Meanwhile, a study from the Montefiore Health System, Bronx (218 patients with cancer, 1,090 patients without cancer) found that patients with cancer had increased odds of death compared to those without cancer (OR 2.45, *p* < 0.001) (2). The authors hypothesized that the socio-economic disadvantage in the Bronx contributed to higher odds of death from COVID-19 in their cohort.

The mortality rate in our combined dataset comprising both patients with and without cancer (36.9%) and in the non-cancer cohort (34.0%) mirrored UK-wide in-hospital mortality from COVID-19 (37%) (9). However, among the cancer cohort, the mortality rate (43.6%) was higher than previously reported by the UKCCMP (28%) or CCC19 (13%) (4, 7). The admission rate to ITU/HDU in the cancer cohort (23.4%) was similar to the national average for all patients hospitalized with COVID-19 (17%) (9). While only one (5.3%) of the patients with metastatic cancer was admitted to ITU/HDU, the mortality rate among those with metastatic disease was not significantly greater than those with less advanced cancer. The higher mortality in our cohort may arise from differences in local ethnic and socio-economic composition, follow-up time, and the burden of co-morbidities among patients attending our specialist unit when compared to the rest of the UK. Our center also accepted capacity transfers from neighboring intensive therapy units, thereby selecting those with more severe COVID-19.

Among the cancer cohort, we found no significant differences in mortality by cancer type or histology. Patients with primary thoracic malignancies did not have an increased risk of COVID-19 mortality compared to all cancers (HR 0.70, 95% CI:0.28–1.8, *p* = 0.46). The mortality rate for these patients (33.3%) matched those of a global COVID-19 and thoracic malignancy database (33%) (14). Interestingly, patients with metastatic disease in our study did not have significantly increased risk of mortality compared to other patients with less advanced cancer.

Meanwhile, SACT within 60 days of a COVID-19 diagnosis was an independent risk factor for mortality in COVID-19 (HR 2.30, 95% CI: 1.16–4.6, *p* = 0.02). Multivariate analysis to delineate the independent risk from cytotoxic chemotherapy or other SACT did not reach significance, probably due to the limited numbers in our cohort. The UKCCMP used multivariate logistic regression adjusting for age, sex, and co-morbidities, finding no statistically significant risk of mortality from COVID-19 with recent cytotoxic chemotherapy or other SACT when compared to patients with cancer who were not receiving treatment (7). From this, the authors concluded that cytotoxic chemotherapy was not associated with an increased risk of mortality. Our results, combining all forms of SACT, suggest that recent SACT predicts mortality from COVID-19. These differences may be accounted for by our longer follow-up time and survival regression. Further complexity is added by the inclusion of hematological malignancies in the UKCCMP study. Interestingly, the UKCCMP noted that palliative chemotherapy was associated with increased adjusted mortality when compared to non-palliative chemotherapy. In our analysis, those on palliative chemotherapy also appeared to have a greater adjusted mortality following COVID-19 (HR 2.29 95% CI: 0.87–6.0, *p* = 0.09) compared to those on neoadjuvant or adjuvant chemotherapy (HR 1.32, 95% CI: 0.30–5.9, *p* = 0.71). This could suggest that chemotherapy particularly predisposes patients with cancer in the palliative setting to death following COVID-19. More solid cancer specific data are needed to confirm or refute these findings.

The strengths of our study include the cohort design, allowing direct comparison of patients with cancer to a matched group of patients without cancer in a single institution. Given the significant geographic variation in outcomes following COVID-19, including only patients from the same center allows us to minimize this effect. We exclude patients with hematological malignancies who have been shown to have poorer outcomes in COVID-19 relative to patients with solid malignancy (1, 2). We collected data on a wide variety of known risk factors and include ethnicity, enabling a more robust multivariate model. Limitations of our study include its retrospective and observational nature. We only included SARS-CoV-2 infection confirmed by RT-PCR on presentation to hospital, potentially omitting many asymptomatic or mildly symptomatic patients. This is particularly true during the early phase of the pandemic as routine surveillance testing was not implemented in the UK. The prioritization and delays to certain cancer treatments may also have introduced bias into our cancer cohort which we are unable to exclude.

In summary, along with known risk factors, cancer and SACT confer an independent risk for mortality following COVID-19. Our findings call for cautious evaluation of the proposition that SACT does not necessarily increase a patient’s risk of dying from COVID-19 (7). They also support the need to continue ‘shielding’ patients with cancer from exposure to the infection. In view of potential resurgences of SARS-CoV-2 infection, prioritization of patients for anti-cancer therapy should take into account pre-morbid risk factors including age, ethnicity, and co-morbidity, rather than potential benefit from anti-cancer therapy alone (6). Discussions with patients should include the benefits of SACT *versus* the potential risks from COVID-19. Our findings in context of others, also raise the need for COVID-19 response policies which are tailored to the local demographic composition. Further studies are urgently needed to fully understand how disparities in socio-economic background, demographics and healthcare practice play a role in modifying the risk profile of cancer and co-morbidity in COVID-19.

## Data Availability Statement

The datasets presented in this article are not readily available because all COVID-19 related data access require permission from the University College London Hospitals Data Access Committee. Requests to access the datasets should be directed to uclh.randd@nhs.net.

## Ethics Statement

In view of the retrospective nature of this study, approval from a Research Ethics Committee (REC) within the UK Health Departments Research Ethics Service and Health Research Authority (HRA) was not necessary. The criterion for REC review exemption was: Research involving previously collected, non-identifiable information.

## Author Contributions

CS, YW, DO, RR, AL, and HS designed and conceived the study. CS, YW, AW, DO, NC, MG, SB, GS, and AL collected the data. CS, YW, AW, RR, AL, and HS analyzed and interpreted the data. CS, YW, and AW performed the statistical analysis. CS, YW, AW, and AL drafted manuscript. DO, AL, and HS provided administrative, technical or material support. HS provided oversight. All authors contributed to the article and approved the submitted version.

## Conflict of Interest

The authors declare the following competing interests: HS has received consulting fees from Novartis, BMS, MSD, Immunocore, Idera, Iovance, Genmab, Sanofi Genzyme/Regeneron, and Macrogenics, Roche. HS has received speaker’s fees from Novartis, BMS, MSD and Sanofi Genzyme. RR reports personal fees from Novartis, personal fees and non-financial support from Daiichi Sankyo, personal fees from Eli-Lilly, personal fees from Pfizer, personal fees and non-financial support from G1Therapeutics, non-financial support from Roche and non-financial support from AstraZeneca. 

The remaining authors declare that the research was conducted in the absence of any commercial or financial relationships that could be construed as a potential conflict of interest.
