# Supplementary material for: Cancer History and Systemic Anti-Cancer Therapy Independently Predict COVID-19 Mortality: A UK Tertiary Hospital Experience
Source: Front Oncol. 2020 Nov 20;10:595804. doi: 10.3389/fonc.2020.595804 (PMC7714940; doi:10.3389/fonc.2020.595804)
Supplement: Supplementary file 1 [file DataSheet_1.docx]

Supplementary Material

# Supplementary Data

**Supplementary Table 1:** Disease characteristics of patients with cancer and SARS-CoV-2 infection. Hazard ratios, 95% confidence intervals (CI) and *p*-values from univariate survival analysis are shown.

|  | **All patients** | **Alive** | **Dead** | **HR (95% CI)** | ***p-value*** |
| --- | --- | --- | --- | --- | --- |
| Total | 94 | 53 | 41 |  |  |
| Male | 62 | 33 (62.3%) | 29 (70.7%) | 1.32 (0.67-2.6) | 0.42 |
| Female | 32 | 20 (37.7%) | 12 (29.3%) | - | - |
| Median age (years) | 71 (62-80) | 66 (58-75) | 78 (71-82) | 1.57 (1.21-2.1)* | <0.001 |
| BMI (kg/m^2^) | 25.1 (21.7 - 30.5) | 26.1 (22.5-31.0) | 24.1(20.4-29.9) | 0.98 (0.93-1.0) | 0.57 |
| Ethnicity |  |  |  |  |  |
| South Asian | 8 | 3 (5.7%) | 5 (12.2%) | 2.07 (0.81-5.3) | 0.13 |
| Black | 6 | 4 (7.5%) | 2 (4.9%) | 0.77 (0.19-3.2) | 0.72 |
| Other | 9 | 4 (7.5%) | 5 (12.2%) | 1.42(0.56-3.6) | 0.46 |
| White | 64 | 36 (67.9%) | 28 (68.3%) | 0.66 (0.33-1.3) | 0.22 |
| Smoking status |  |  |  |  |  |
| Ex- or active smoker | 49 | 27 (50.9%) | 22 (53.7%) | 1.25 (0.65-2.4) | 0.50 |
| Co-morbidities |  |  |  |  |  |
| Cardiovascular disease | 18 | 8 (15.1%) | 10 (24.4%) | 1.43 (0.70-2.9) | 0.33 |
| Dementia | 7 | 4 (7.5%) | 3 (7.3%) | 0.85 (0.26-2.7) | 0.78 |
| Diabetes | 24 | 17 (32.1%) | 7 (17.1%) | 0.57 (0.25-1.3) | 0.17 |
| Congestive cardiac failure | 9 | 6 (11.3%) | 3 (7.3%) | 0.62 (0.19-2.0) | 0.43 |
| Liver disease | 3 | 2 (3.8%) | 1 (2.4%) | 0.63 (0.09-4.6) | 0.65 |
| Hypertension | 37 | 16 (30.2%) | 21 (51.2%) | 2.24 (1.21-4.1) | 0.01 |
| Peripheral vascular disease | 2 | 1 (1.9%) | 1 (2.4%) | 1.39 (0.19-10.1) | 0.75 |
| Cerebrovascular disease | 12 | 3 (5.7%) | 9 (22.0%) | 2.84 (1.34-6.0) | 0.006 |
| Chronic lung disease | 14 | 7 (13.2%) | 7 (17.1%) | 1.19 (0.53-2.7) | 0.68 |
| Chronic kidney disease | 12 | 5 (9.4%) | 7 (17.1%) | 1.95 (0.86-4.4) | 0.11 |
| Ongoing corticosteroid therapy | 4 | 3 (5.7%) | 1 (2.4%) | 0.49 (0.07-3.6) | 0.48 |

*per 10 year increase in age.

**Supplementary Table 2:** Univariate analysis of cancer-specific factors in patients with SARS-CoV-2 infection.

|  | **Total** | **Alive** | **Dead** | **HR (95% CI)** | ***p*-value** |
| --- | --- | --- | --- | --- | --- |
| Active cancer | 58 | 32 (60.4%) | 26 (63.4%) | 1.12 (0.59-2.11) | 0.74 |
| Metastatic cancer | 19 | 10 (18.9%) | 9 (22.0%) | 1.13 (0.54-2.37) | 0.75 |
| Cancer type |  |  |  |  |  |
| Genitourinary | 24 | 11 (20.8%) | 13 (31.7%) | 1.65 (0.85-3.19) | 0.14 |
| Gastrointestinal | 23 | 12 (22.6%) | 11 (26.8%) | 1.04 (0.52-2.07) | 0.92 |
| Thoracic | 15 | 10 (18.9%) | 5 (12.2%) | 0.70 (0.28-1.79) | 0.46 |
| Female genital tract | 9 | 6 (11.3%) | 3 (7.3%) | 0.60 (0.19-1.96) | 0.40 |
| Breast | 8 | 4 (7.5%) | 4 (9.8%) | 1.56 (0.55-4.38) | 0.40 |
| Other | 15 | 10 (18.9%) | 5 (12.2%) | 0.66 (0.26-1.69) | 0.39 |
| Histology |  |  |  |  |  |
| Adenocarcinoma | 41 | 20 (37.7%) | 21 (51.2%) | 1.38 (0.73-2.62) | 0.32 |
| Squamous cell carcinoma | 16 | 8 (15.1%) | 8 (19.5%) | 0.98 (0.45-2.13) | 0.95 |
| Other | 27 | 18 (34.0%) | 9 (22.0%) | 0.68 (0.32-1.44) | 0.31 |
| Data missing | 10 | 7 (13.2%) | 3 (7.3%) | - | - |
| Cancer treatment |  |  |  |  |  |
| All systemic anti-cancer therapy | 25 | 12 (22.6%) | 13 (31.7%) | 1.42 (0.74-2.75) | 0.29 |
| All chemotherapy | 15 | 8 (15.1%) | 7 (17.1%) | 1.03 (0.45-2.32) | 0.95 |
| Palliative chemotherapy | 8 | 3 (5.7%) | 5 (12.2%) | 1.53 (0.60-3.90) | 0.37 |
| Neoadjuvant/ adjuvant chemotherapy | 7 | 5 (9.4%) | 2 (4.9%) | 0.58 (0.14-2.40) | 0.45 |
| Endocrine therapy | 8 | 3 (5.7%) | 5 (12.2%) | 2.43 (0.95-6.21) | 0.06 |
| Targeted anti-cancer therapy | 2 | 1 (1.9%) | 1 (2.4%) | 1.35 (0.18-9.82) | 0.77 |
| Immunotherapy | 4 | 2 (3.8%) | 2 (4.9%) | 1.11 (0.27-4.62) | 0.88 |
| Radiotherapy | 5 | 3 (5.7%) | 2 (4.9%) | 1.00 (0.24-4.16) | 1.00 |
| Surgery | 3 | 2 (3.8%) | 1 (2.4%) | 0.58 (0.08-4.23) | 0.59 |
